# Trajectories of Body Composition during Advanced Aging in Consideration of Diet and Physical Activity: A 20-Year Longitudinal Study

**DOI:** 10.3390/nu12123626

**Published:** 2020-11-25

**Authors:** Alexandra Jungert, Gerrit Eichner, Monika Neuhäuser-Berthold

**Affiliations:** 1 Institute of Nutritional Science, Justus Liebig University, Goethestr. 55, D-35390 Giessen, Germany; alexandra.jungert@ernaehrung.uni-giessen.de; 2 Interdisciplinary Research Center for Biosystems, Land Use and Nutrition (IFZ), Justus Liebig University, Heinrich-Buff-Ring 26-32, D-35392 Giessen, Germany; 3 Mathematical Institute, Arndtstr. 2, Justus Liebig University, D-35392 Giessen, Germany; gerrit.eichner@math.uni-giessen.de

**Keywords:** body composition, anthropometric data, longitudinal changes, aging, physical activity, protein intake

## Abstract

This prospective study investigates age-dependent changes in anthropometric data and body composition over a period of two decades in consideration of physical activity and diet in community-dwelling subjects ≥60 years. Overall, 401 subjects with median follow-up time of 12 years were examined. Fat-free mass (FFM) and fat mass (FM) were analyzed using bioelectrical impedance analysis. Physical activity was assessed via a self-administered questionnaire. Dietary intake was examined by 3-day dietary records. Linear mixed-effects models were used to analyze the influence of age, sex, physical activity and energy/protein intake on anthropometric data and body composition by considering year of entry, use of diuretics and diagnosis of selected diseases. At baseline, median values for daily energy and protein intakes were 8.5 megajoule and 81 g and physical activity index was 1.7. After adjusting for covariates, advancing age was associated with parabolic changes indicating overall changes from age 60 to 90 years in women and men in body mass: −4.7 kg, −5.0 kg; body mass index: +0.04 kg/m^2^, −0.33 kg/m^2^; absolute FFM: −2.8 kg, −3.5 kg; absolute FM: −1.8 kg, −1.2 kg and waist circumference: +16 cm, +12 cm, respectively. No age-dependent changes were found for upper arm circumference and relative (%) FFM. Dietary and lifestyle factors were not associated with changes in anthropometric or body composition parameters. In summary, the results indicate non-linear age-dependent changes in anthropometric data and body composition, which are largely unaffected by the degree of habitual physical activity and dietary protein intake in well-nourished community-dwelling subjects.

## 1. Introduction

Advancing age comes along with deteriorations in health status, which have been linked to changes in body composition [1,2,3]. Biological aging is associated with characteristic changes in body mass and body composition [1,4,5,6,7,8,9,10,11,12,13,14]. Fat-free mass (FFM) [10], especially muscle mass [1,13,14], usually decreases in older adults, while fat mass (FM) increases [10] and redistributes from peripheral to abdominal body areas [4,9]. Such changes may lead to sarcopenic obesity, which has been associated with an increased risk for morbidity, mortality, health care costs and impaired quality of life [15,16]. The extent to which these age-related changes in body composition depend on genetic programming and on lifestyle factors, such as diet and physical activity, is under discussion [10,15].

Lifestyle factors are of particular interest as these are modifiable risk factors. Some studies suggest that regular physical activity [7,8,17,18] and an adequate protein intake [19,20,21,22] may protect against an unintended weight loss or slow down the age-dependent decline in physical function and skeletal muscle mass. Higher protein intake levels are often recommended for elderly people compared to young- and middle-aged adults due to anabolic resistance and catabolic situations caused by underlying diseases [23]. On the other hand, an impact of lifestyle factors on body composition or physical performance in older adults was repeatedly not found [24,25,26,27]. Thus, it is a moot point whether and to what extent older subjects can proactively influence their body composition in the course of aging by their own lifestyles.

Age-related changes in anthropometric data and body composition were frequently investigated by cross-sectional approaches. However, to scrutinize intra-individual changes in body composition in consideration of influencing factors longitudinal studies are mandatory [28]. Available long-term studies often had a mean follow-up period ≤10 years or were not exclusively on older subjects, investigated relatively small samples, focused only on anthropometric data, collected data only twice or provided no data on physical activity and dietary intake [1,4,5,6,7,8,9,10,11,12,13,14]. Thus, knowledge on the trajectory and the extent of age-related changes in body mass and body composition, as well as on potential influencing factors, such as diet and physical activity, is still limited.

The present study therefore aims to analyze non-linear age-related changes in parameters of anthropometry and body composition in community-dwelling subjects ≥60 years based on multiple follow-ups over two decades by considering habitual physical activity and diet, as well as effect modifications by sex, year of study entry, presence of chronic diseases and lifestyle factors.

## 2. Subjects and Methods

### 2.1. Study Design

This exploratory investigation is based on the longitudinal study on nutrition and health status in senior citizens in Giessen, Germany (GISELA study). Detailed descriptions of the study design and methods can be found in the literature, e.g., [29,30,31]. This study was initiated in 1994 and was conducted in the Institute of Nutritional Science at the Justus Liebig University of Giessen, Germany. Data on body composition, physical activity, nutrition and health status were obtained annually until 1998 and biennially thereafter. Physical examinations, including measurements of anthropometric data and body composition, were done in the morning hours after an overnight fast in the Institute of Nutritional Science between July and October. Inclusion criteria were age of at least 60 years, physical mobility and residence in Giessen or the surrounding area.

This study was conducted according to the guidelines laid down in the Declaration of Helsinki and was approved by the Ethical Committee of the Faculty of Medicine at the Justus Liebig University of Giessen, Germany (Project identification code A 30/95). Participants provided informed consent.

### 2.2. Study Subjects

**Figure 1:**
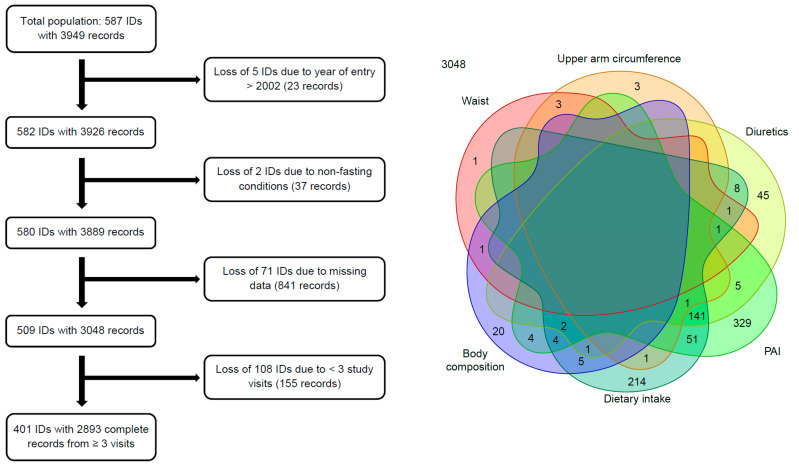
Reasons for losing or excluding subjects from the analyses. **Left**: This flowchart displays the numbers and reasons for losing or excluding subjects (indicated by ID) and/or records. **Right**: The Venn diagram states more precisely the sources and numbers of losses of the 841 incomplete data records. Each colored shape represents the set of records, which have a missing value in the variable naming the shape. The inscribed numbers are the counts of those records. A model variable that does not appear here has no missing values at all. PAI, physical activity index.

From 1994 to 2006, 587 volunteers were recruited, but not all subjects participated in each follow-up. Reasons for loss to follow-up over the entire study period were illness (37%), no interest (18%), death (16%), no accessibility (12%), relocation (4%) and miscellaneous (13%). For the present analysis, subjects with complete data records on at least three visits between 1994 and 2014 were considered. Since only five subjects joined the study after 2002 and the year of entry was a cofactor in the analyses, those five subjects were not considered for the present analyses. Figure 1 illustrates the numbers and reasons for losing or excluding subjects and records, respectively.

Data from 401 subjects (278 women and 123 men) were analyzed. On average, these participants visited the institute seven times within the study period (range: 3–13 visits). Consequently, the median follow-up time was twelve years (range: 2–20 years).

### 2.3. Anthropometric Data

After shoes, coats and sweaters had been removed, body mass and height of the participants were assessed by trained staff with a calibrated digital scale with stadiometer (Seca, Vogel & Halke GmbH & Co. KG, Hamburg, Germany). Based on the mass of remaining clothes, 0.5 to 1.0 kg was subtracted from the measured body mass. Body mass index (BMI) was calculated by dividing body mass in kg by the square of body height in meter. Waist circumference was measured with a tape, while standing in an upright position, across the narrowest point between the lower rib and the iliac crest. Mid-upper arm circumference was determined with a tape, while standing in an upright position, on the upper arm halfway between the acromion process and the olecranon process.

### 2.4. Body Composition

Body composition was investigated in a supine position by trained staff using a single frequency (50 kHz) bioelectrical impedance analyzer (Akern-RJL BIA 101/S, Data Input, Frankfurt, Germany). Electrodes were placed on foot and hand after skin surfaces were cleaned with alcohol. FFM and FM were calculated by applying the equation from Roubenoff et al. [32]. The mean coefficients of intra-day variations for 20 repeated measurements in seven subjects aged 22–35 years were 1.0% ± 0.3% and 1.6% ± 0.7% for resistance and reactance, respectively.

### 2.5. Physical Activity Index

At baseline and in each follow-up, subjects were asked about their weekly physical activity patterns by a self-administered questionnaire. Energy expenditures of the different activities were calculated using multipliers for resting metabolic rate according to the WHO [33], as described in detail elsewhere [29]. For each subject, the sum of energy expenditures for the different activities was calculated and added to the measured resting metabolic rate. The resting metabolic rate was assessed by an open-circuit indirect calorimeter (Deltatrac^TM^ MBM-100, Hoyer, Bremen, Germany) [30]. The physical activity index (PAI) of each subject was calculated by the ratio of total energy expenditure to resting metabolic rate. The PAI values can be interpreted as sedentary (1.0–1.4), low active (1.4–1.6), active (1.6–1.9) and very active (1.9–2.5) lifestyle [34].

### 2.6. Energy and Protein Intake

At baseline and in each follow-up, on three consecutive days subjects completed a self-administered estimated dietary record, which included 146 food items for which typical household measures (e.g., cup, spoon, slice) and weights were given. Energy and protein intakes were calculated using the German Food Code and Nutrient Database version 3.02 (Max Rubner-Institute, Karlsruhe, Germany). In this context, energy intake via dietary fibers was taken into account. The dietary record was validated by comparing the calculated nitrogen intake using a former German Food Code and Nutrient Database version with 24 h urine nitrogen excretion and the calculated energy intake with the assessed basal metabolic rate as described previously [31].

### 2.7. Use of Diuretics and Diagnosis of Selected Diseases

Subjects were asked about their use of diuretics by self-administered questionnaires. Their respective answers were subsequently summarized by a dichotomous variable (no vs. yes), which was considered as potential confounding factor as the use of diuretics might especially affect BIA measurement results [35].

Subjects filled out questionnaires on the presence of diseases diagnosed by their physicians. For the following investigation, a summarizing dichotomous variable was derived combining all information on the presence of diseases that are assumed to affect body composition or measurement methods, such as cancer, osteoporosis, thyroid disease, diabetes, heart disease and chronic inflammatory bowel/liver/kidney diseases. Consequently, this dichotomous variable indicated the history of at least one of the above-mentioned diseases for each participant.

### 2.8. Statistical Analyses

For statistical analyses and graphics, the open-source software R version 3.6.1 (2019-07-05) [36] and 4.0.2 (2020-06-22) including the packages venn [37], Gmisc [38], lattice [39], car [40], effects [40], MANOVA.RM [41], lme4 [42] (with support from [43,44,45]), pbkrtest [46] and multcomp [47] were used. Results were considered statistically significant when *P* values were below 0.05. In linear mixed-effects models, adjustments for simultaneous inference (for both tests and confidence intervals) were performed using methods for general linear hypotheses [47].

Baseline data were expressed as median and 25th and 75th percentiles for the entire study population and stratified by sex. Comparison of the excluded participants with the sample of 401 selected participants with respect to their baseline data (body mass, body height, BMI, waist circumference, upper arm circumference, relative FFM, absolute FM, age, PAI, energy intake, protein intake) was performed with one-factorial multivariate ANOVA (MANOVA). Absolute FFM had to be excluded from this comparison because of its collinearity to other variables. This analysis required complete baseline data for all of the above 11 variables. Consequently, only 97 of the excluded subjects could be considered, while 89 of the excluded subjects had to be ignored in this comparison. In addition, univariate comparisons were performed for all of those variables (including absolute FFM).

Prior to the main analyses, the potential changes in lifestyle factors during advanced aging were investigated using linear mixed-effects models with sex, a linear and quadratic function of age (scaled to decades and centered around 7.2) and the interaction between sex and age as fixed effects, and subject and linear age as random effects.

Metric parameters of anthropometry (body mass, BMI, waist circumference and upper arm circumference) and body composition (relative and absolute FFM, as well as absolute FM) functioned as outcome variables and were analyzed in separate linear mixed-effects models. At first, the focus was on the age-related changes in parameters of anthropometry and body composition without including other potentially influencing or confounding variables. Based on the literature [10], curvilinear age-related changes in anthropometric and body composition parameters were assumed. Therefore, linear mixed-effects models with a linear and quadratic function of individual age (scaled to decades and centered around 7.2) as the only fixed effects, i.e., without other covariables, were set up initially. These models were then extended by fixed effects for sex (female vs. male), PAI (scaled to two-digit numbers by dividing by 0.1 and then centered around 16) and protein intake (scaled to decagram/d by dividing by 10 and then centered around 6.2). In the model for waist circumference, body mass (centered around 70) was included as additional fixed effect. The year of entry (centered around 1996) and the use of diuretics (no vs. yes) were included in each model as confounding variables. In addition to the aforementioned main fixed effects, the following interaction fixed effects of sex and age were considered in these models: sex with age (linear, quadratic), PAI and protein intake, as well as linear age with PAI, year of entry and protein intake. Centering and scaling the metric covariables were performed to reduce potential multicollinearity between model terms and to increase the numerical stability of the computational fitting process. The latter was in addition checked using up to seven different numerical optimizers and various convergence criteria. To facilitate the interpretation of the results, the coefficient estimates (CE) and their 95% confidence intervals (95% CI) were rescaled.

The linear mixed-effects models comprised three random effects with an unrestricted covariance structure to account for the longitudinal and hence correlated data structure within individuals on the one hand, and for the inter-individual variability in age-trends on the other hand: subject, linear age and quadratic age. In the models with the response variables waist circumference, upper arm circumference or absolute FFM, the covariance structure of the random effect vectors was restricted by setting correlations between squared age term and intercept and linear age term to zero to achieve adequate convergence of the model fitting process. The restricted maximum likelihood method was used to estimate variance components. Missing values were considered to be missing completely at random. Residual diagnostics for the fitted models by means of normal qq-plots indicated moderately heavier tails for the residuals than appropriate under the normality assumption for the errors. However, with regard to the relatively large sample size of 2893 records, the inferential results (*P*-values and confidence intervals) were considered as reliable due to the asymptotic distributional properties of the model estimators. Nevertheless, sensitivity analyses were performed in which outliers were excluded (details see below).

The following sensitivity analyses were conducted:

(1) Each initial model, which included only the age effects, was compared with its corresponding full model including all random and fixed effects using the Kenward-Roger method [46].
(2) All model-fits and analyses were repeated without model-wise detected unduly influential observations (based on Cook distances, i.e., >1) and outliers (based on Bonferroni-adjusted *P*-values (<0.05) from testing each observation in turn to be a mean-shift outlier with respect to the Studentized residuals). For each model in turn the detected “outlying” observations were eliminated. This could result in a slight reduction of the sample size, if a subject was left with fewer than three complete data records.
(3) Whether a history of selected diseases influenced age-related changes in anthropometric and body composition data was investigated by including two additional fixed effects: disease diagnosis (no vs. yes) and the interaction between disease diagnosis and linear age.
(4) All full models were rebuilt with protein intake being replaced by total energy intake (centered around 9.1 megajoule (MJ) per day) to study, whether the inclusion of energy intake affects the results.
(5) Finally, the analyses were repeated with the restriction to subjects with complete data records on at least seven visits (leaving 226 individuals with 2087 records) to investigate the robustness of the findings with respect to the number of follow-ups.

## 3. Results

### 3.1. Baseline Characteristics

The descriptive characteristics of the GISELA subjects at baseline are presented in Table 1. The results of the MANOVA indicate significant differences between selected and excluded participants with regard to their baseline data (*P* < 0.001) as illustrated in Supplementary Figure S1. Univariate one-factorial ANOVAs suggest that selected subjects were younger (*P* < 0.001) and taller (*P* = 0.012). No differences are found in the other variables (all *P* > 0.05).

### 3.2. Age-Related Changes in Lifestyle Factors

The age-related changes in PAI and intakes of energy and protein adjusted for sex are illustrated in Supplementary Figure S2. PAI decreases in the course of aging (linear age effect: *P* < 0.001; quadratic age effect: *P* = 0.029), whereas energy intake remains on the same level (both age effects: *P* > 0.05) and protein intake increases over time (linear age effect: *P* = 0.003; quadratic age effect: *P* = 0.004). Although male sex is associated with a slightly lower PAI (*P* = 0.040) and higher energy and protein intake (*P* < 0.001), the age-related changes in these parameters are not significantly modified by sex (all *P* > 0.05). Similar results were found after the exclusion of model-wise unduly influential observations and outliers.

### 3.3. Age-Related Changes in Anthropometry and Body Composition before Adjusting for Cofactors

The age-related changes in parameters of anthropometry and body composition before consideration of potential influencing factors are shown in Table 2. All parameters alter in the course of aging apart from upper arm circumference. The observed associations possess a non-linear character with waist circumference being the only parameter showing a virtually continuous age-related increase.

### 3.4. Age-Related Changes in Anthropometry and Body Composition by Considering Cofactors

**Figure 2:**
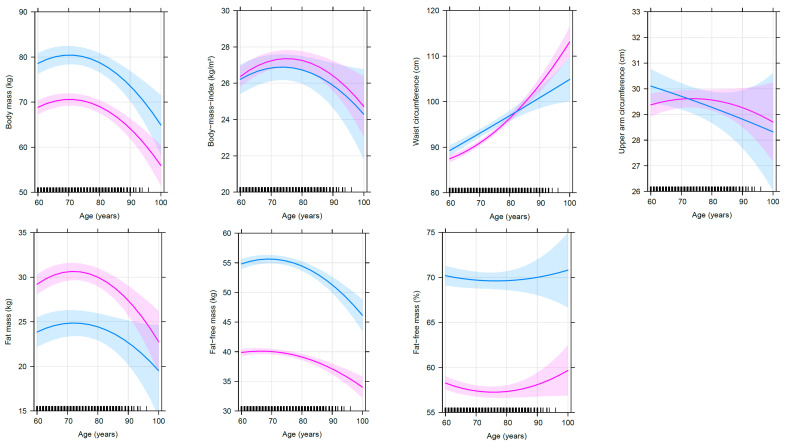
Age-related changes in anthropometric and body composition parameters separated by sex. This figure illustrates the selected effects of age and sex on anthropometric and body composition parameters after controlling for other variables in the linear mixed-effects models (*n* = 401). The thick lines represent the estimated means and the respective colored areas reflect the 95% confidence intervals. The associations are illustrated in magenta and blue color for female (*n* = 278) and male (*n* = 123) subjects, respectively. The small black pillars on the *x*-axis reflect the numbers of records at the respective age of the subjects.

The results of the linear mixed-effects models after taking potential influencing factors into account are shown in Table 3 for anthropometric data and in Table 4 for body composition parameters. Advancing age from 60 to 90 years is associated with parabolic, predominantly concave changes in body mass, BMI, absolute FFM, absolute FM and waist circumference. From the age of 60 to 90 years, an average person of the present study population loses eventually −4.7 kg (women) and −5.0 kg (men) body mass, −2.8 kg (women) and −3.5 kg (men) absolute FFM, −1.8 kg (women) and −1.2 kg (men) absolute FM and shows slight changes in BMI of +0.04 kg/m^2^ (women) and −0.33 kg/m^2^ (men) and—in contrast—a substantial increase in waist circumference of +16 cm (women) and +12 cm (men). Figure 2 illustrates that body mass slightly increases and absolute FFM remains nearly stable until the age of 70 years and then starts to decline with advancing age. Absolute FM and BMI both increase until 73 and 75 years of age, respectively, and after a short plateau phase the parameters decrease as well. In contrast, waist circumference shows an age-related apparently accelerating increase with no sign of flattening. No age-dependent changes are found for relative FFM and upper arm circumference after the consideration of relevant cofactors. Referring to the 95% CI, the variability is greater for absolute FM, BMI and relative FFM compared to absolute FFM and waist circumference, especially at higher ages.

**Figure 3:**
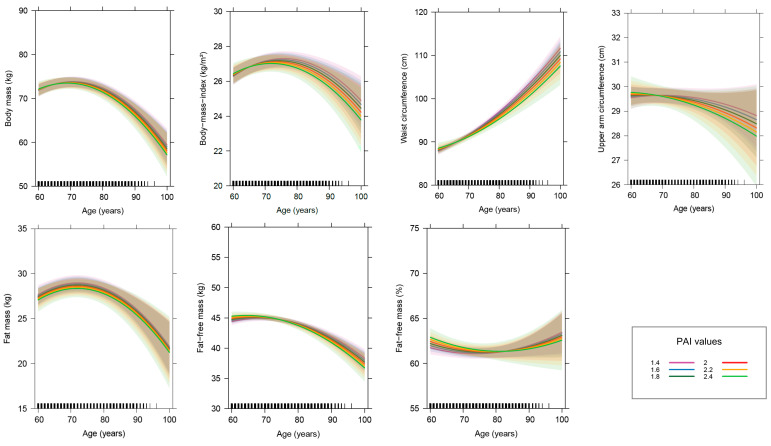
Age-related changes in parameters of anthropometry and body composition in dependence of the physical activity index (PAI). This figure displays the effect of age on parameters of anthropometry and body composition in dependence of the PAI after controlling for other variables in the linear mixed-effects models (*n* = 401). The colored lines show the age-related changes in parameters of anthropometry and body composition at different PAI values, ranging from sedentary/low active to very active lifestyles. The thick lines represent the estimated means and the respective colored areas reflect the 95% confidence intervals. The small black pillars on the *x*-axis reflect the numbers of records at the respective age of the subjects.

**Figure 4:**
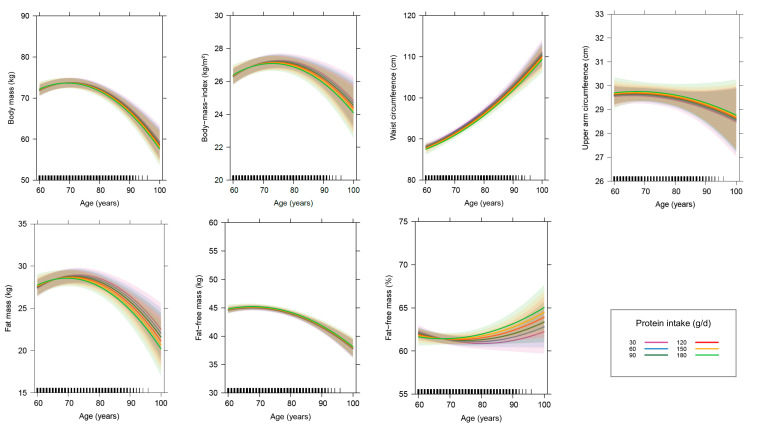
Age-related changes in parameters of anthropometry and body composition in dependence of the daily protein intake. This figure displays the effect of age on parameters of anthropometry and body composition in dependence of the daily protein intake after controlling for other variables in the linear mixed-effects models (*n* = 401). The colored lines show the age-related changes in parameters of anthropometry and body composition at different dietary protein intake levels, ranging from 30 to 180 g/day. The thick lines represent the estimated means and the respective colored areas reflect the 95% confidence intervals. The small black pillars on the *x*-axis reflect the numbers of records at the respective age of the subjects.

Male sex is associated with higher body mass, waist circumference and FFM (absolute and relative), whereas female sex is associated with a higher absolute FM. Furthermore, male subjects show a smaller increase in waist circumference with advancing age. PAI and daily protein intake exhibit no significant effects on variables of anthropometry and body composition or on the age-related changes in these variables (Figure 3 and Figure 4). The year in which subjects joined the study is of relevance for all dependent variables except for relative FFM. Subjects who joined the study later have significantly higher body mass, upper arm circumference, absolute FFM and FM, as well as by trend a higher BMI. Furthermore, the decline in absolute FFM and the increase in waist circumference with advancing age are stronger in subjects, who joined the study later (Supplementary Figure S3). The use of diuretics is by trend associated with a lower relative FFM, whereas body mass is a significantly positive predictor for waist circumference.

### 3.5. Sensitivity Analyses

#### 3.5.1. Relevance of Other Factors Besides Advancing Age

The comparison of the full model including all fixed effects with a reduced model including only linear and quadratic age effects confirms an improvement of the model fit for all investigated response variables (all *P* ≤ 0.01), except for BMI (*P* = 0.10).

#### 3.5.2. Analyses without Unduly Influential Observations and Outliers

After the exclusion of model-wise unduly influential observations and outliers, the results remain unchanged, except for the effect of the use of diuretics, which reaches the significance level after the exclusion (Supplementary Tables S1 and S2).

#### 3.5.3. Analyses with the History of Chronic Diseases as Additional Fixed Effect

When the history of chronic diseases is integrated in the model in form of an additional fixed effect, the results remain largely unchanged except for the linear age effect on BMI and the interaction between sex and age for waist circumference; both fixed effects lose significance (Supplementary Tables S3 and S4). The disease history shows no direct effect on any dependent variable.

#### 3.5.4. Analyses with Different Dietary Factors as Fixed Effect

The replacement of daily protein intake by total energy intake yields comparable results as regards the identified predicting variables (Supplementary Tables S5 and S6). Only the significance of the linear age effect on BMI vanishes when protein intake is replaced by energy intake. Energy intake shows no association with anthropometric or body composition data.

#### 3.5.5. Analyses Restricted to Subjects with at Least Seven Complete Data Records

The restriction of the initial analyses to subjects with at least seven complete data records (median follow-up time is 15 years, range: 8–20 years) leads to following changes in the results: for upper arm circumference, a significant interaction between year of entry and linear age is found, although the linear age effect itself is still not significant. For waist circumference, the sex effect, as well as the interaction between sex and age, lose significance. For absolute FFM, the significant effect of the year of entry and its interaction with age vanish. The other results remain substantially unchanged (Supplementary Tables S7 and S8).

## 4. Discussion

The unique feature of this longitudinal study on changes in anthropometric and body composition parameters in older adults is its duration over two decades during which multiple follow-ups were performed, including assessments of dietary intake and physical activity. In addition to what is already known from previous studies, the present study contributes data on the trajectory of changes in body mass, waist circumference and parameters of body composition in the light of age-related changes in physical activity and nutritional behavior for both women and men, with a focus on the advanced age. The main results show an increase in body mass, BMI and FM until the age of approximately 70 and 75 years, after which these parameters start to decrease. Absolute FFM seems to remain stable until 70 years of age and then decreases. Relative FFM remains fairly stable over time. However, in subjects with at least seven complete data records, a trend for an age-related decline in relative FFM becomes evident, indicating a more rapid decline in FFM in comparison to FM in the long-term. The wide scattering of the trajectory of changes in relative FFM after the age of 80 years points towards very individual age-related changes in body composition, which broadly being caused by highly individual changes in absolute FM. Waist circumference is the only parameter showing a steady increase with advancing age. Neither dietary factors nor physical activity affected these age-related changes.

The results also demonstrate that age-related changes in body composition may be missed when longitudinal data are linearly investigated. Likewise, the assessment of only two sampling points falls too short to accurately reflect the dynamics of such changes. This is in line with the few available long-term studies [7,8,10], which investigated non-linear age-related changes in body composition in either smaller sample sizes or only in male populations by considering proxies for physical activity but not for dietary changes.

Some studies found sex-specific differences in age-dependent changes in anthropometric data and body composition [5,13,48,49]. In the present study, such differences were evident only for waist circumference, indicating a stronger increase in women than in men. Moreover, the sexual dimorphism in waist circumference phenotype diminished with advancing age, as indicated by the sensitivity analysis in subjects with at least seven visits. In this context, one has to consider that men show more often an android fat distribution already in middle age compared to women [50], while women experience age-related hyperkyphosis more often than men [51]. The latter might result in a higher waist circumference due to a curved back and thus reflect a limitation of the measuring method. Based on the present results, anthropometric and body composition variables differ by sex, with the exception of BMI and upper arm circumference, whereas the changes in anthropometric and body composition data along the age axis differ only marginally between sexes.

In this study, age-related changes in anthropometric and body composition parameters were not affected by physical activity or dietary intake, which is in line with some previous findings from longitudinal studies with shorter observation periods [25,26,27]. The impact of physical activity on body composition during aging may depend on the type and intensity of the physical activity. In the GISELA study, the main activities of the subjects are gardening, housework and walking, while time spend with sport activities is relatively minor [30]. Other studies demonstrated that subjects with low fitness levels at baseline lost significantly more FFM than very fit subjects [17] and resistance training was capable to preserve FFM under energy restriction in older women [18]. In one study over eight years of follow-up, moderately active subjects ≥65 years showed a smaller decrease in body mass than inactive subjects, but higher physical activity provided no substantial additional benefit [52]. The GISELA subjects showed a relatively active lifestyle and in this respect the study cohort is quite homogeneous, what could explain the lacking impact of physical activity.

The turning points when parameters of body composition start to decline with advancing age seem to occur somewhat later in the GISELA subjects compared to those observed in one longitudinal study in males aged 20 to 96 years [10]. These discrepancies may be partly due differences in the study population and methods employed. They could also point to a certain plasticity of the trajectory of age-related changes in body composition, which may be influenced by lifestyle and/or environmental factors. As no impact of PAI and protein/energy intake was found, this finding might be a result of the overall fitness level, the relatively high protein intake (which was on average above the current reference value) and the sufficient energy intake. It should be emphasized that in the GISELA subjects, energy intake remains stable over the course of aging, while protein intake (g/day) increases and PAI attenuates only slightly between the age of 60 and 90 years from around 1.7 to 1.6. Even if PAI is not associated with changes in body composition in this cohort, it cannot be ruled out that a high PAI may positively impact muscle strength, as shown in some interventional studies [53,54].

The age-related changes in body mass and absolute FM show similar curve shapes, but absolute FM reaches the maximum later than body mass. Both parameters suggest that FM decreases in very advanced age, but provide no information on the redistribution of fat tissue from peripheral to abdominal regions, as indicated by waist circumference. Overall, the observed reduction in FFM accompanied by an increase in abdominal fat tissue in advancing age increases the risk for disability, functional impairment [55,56] and a state of low-grade inflammation [57] and thus may contribute to multimorbidity in older age, which in turn may affect energy balance of older subjects [58]. Existing diseases may boost or cause age-related changes in body composition. In the present study, the observed age-related changes occurred independently of the presence of relevant chronic diseases. This finding corresponds to a 3-year study in Chinese ≥ 70 years [48]. Thus, age *per se* seems to be a major determinant for changes in body mass and body composition in older subjects. However, to study the effect of diseases in depth, it is essential to consider the severity, duration and medication, and thus would need a more differentiated approach.

In very advanced age, accelerated loss of body tissue as well as abdominal obesity are apparently overriding health issues rather than a higher prevalence of generalized obesity [48]. Research should elucidate what triggers the turning point occurring between the ages of 70 and 80 years and what are the key influencing factors in order to identify suitable measures to maintain a healthy body mass and composition or at least to slow down the losses. The present results indicate that an active lifestyle in combination with adequate intakes of energy and protein may not be sufficient to prevent age-related changes in body composition in community-dwelling older subjects.

Some intervention studies suggest that, in particular, resistance exercise may counteract the age-related losses of muscle mass and strength [59]. Under the assumption that an increased supply with dietary protein and/or specific amino acids, such as leucine, may stimulate muscle protein synthesis, nutritional interventions with various protein sources, intake levels and regimens are also under discussion for preventing age-related losses of muscle and thus of lean body mass [16]. Whether and which specific exercise programs and/or dietary regimen, either individually or combined can be suitable and practicable measures to prevent or mitigate the age-related changes in both FM and FFM in the long-term remains to be shown. For the successful development of targeted strategies, a comprehensive understanding of the mechanisms involved in the changes of body composition during advanced aging is required.

Because neither physical activity nor energy and protein intake had a significant impact on the trajectory of body mass, FM and FFM along aging in the present cohort, the turning point observed at the age between 70 and 80 years may reflect an increase in catabolic mechanisms. To which extent this shift in the metabolic balance may be a consequence of the increasing manifestation of diseases and/or related changes in intrinsic mechanisms of aging, requires further exploration. It is well known that several hormones, such as growth hormone [60], sex steroids [16,61], thyroid hormones [62] and adipokines [16], as well as inflammatory processes [16] and the immune system [63] may affect either body composition or energy expenditure. In this context, the possible influence of age-related changes in the neuroendocrine-immune system may deserve special consideration [16].

The interpretation of the findings should consider the following limitations. As this study is based on a convenience sample of senior citizens, the results may not be generalizable. Selection bias may have occurred over time, as indicated by differences in body height and age between investigated subjects and those who were lost by follow-up or excluded due to missing data. Thus, associations may have been underestimated. The observed increase in anthropometric and body composition parameters in dependence of the first examination year of the participants might indicate a secular effect [10]. Data on disease history, physical activity and dietary intake relied on self-administered questionnaires and estimated dietary records. The metabolic equivalents used to calculate the PAI may not correspond to the actual intensity level in older adults [7]. Distinctions between types and severities of diseases as well as analyses on the incident of diseases over time were not performed. Finally, body composition was assessed by BIA, which has limitations [35]. However, a BIA equation was applied, which has been validated against dual-energy X-ray absorptiometry in older adults [32]. For the strengths, it may be considered that this study included multiple follow-ups over two decades on both physical activity and dietary intake. Body composition and lifestyle factors were assessed by using the same devices and methods over the entire study period. Assessment of physical activity was based on multiples of measured resting energy expenditure. For the dietary assessment, a tool especially developed for the GISELA study was used, which was validated for protein and energy intake. Non-linear effects as well as effect modifications were investigated, sensitivity analyses were performed and simultaneous inference procedures were applied.

## 5. Conclusions

The results indicate parabolic, predominantly concave age-dependent changes in anthropometric data and body composition, which appear largely unaffected by the degree of habitual physical activity and protein or energy intake in community-dwelling older adults. With increasing age, the decline in body mass, BMI, FFM and FM accelerates even in physically active subjects with on an average sufficient protein and energy intake. Facing the increasing life expectancy, future research should elucidate what triggers the points of descent of the parabolic trends occurring at an age between 70 and 80 years and the progressive decline of FM and FFM thereafter.

## Figures and Tables

**Table 1:** Characteristics of the GISELA subjects at baseline.

Variable	Total (*n* = 401)			Women (*n* = 278)			Men (*n* = 123)		
	Median	P25	P75	Median	P25	P75	Median	P25	P75
Age (years)	66	62	70	67	62	71	66	63	70
Body height (cm)	164	159	171	162	158	165	174	170	178
Body mass (kg)	72	64	80	68	62	77	79	73	87
Body mass index (kg/m^2^)	26	24	29	26	24	29	26	24	28
Waist circumference (cm)	90	83	97	87	81	94	96	91	103
Upper arm circumference (cm)	30	28	31	29	28	31	30	28	31
Fat mass (kg)	27	23	33	28	24	35	23	19	29
Fat-free mass (kg)	42	38	52	39	38	42	54	53	58
Fat-free mass (%)	61	56	67	58	54	62	70	67	73
Physical activity index	1.7	1.6	1.8	1.7	1.6	1.8	1.7	1.6	1.8
Energy intake (MJ/day)	8.5	7.1	10	8.1	6.8	9.6	9.9	8.0	12
Protein intake (g/day)	81	65	96	75	62	92	86	74	103
	***n***	**%**		***n***	**%**		***n***	**%**	
Female sex	278	69							
Use of diuretics	42	11		32	12		10	8.1	
Year of entry after 1996	126	31		95	34		31	25	
Disease diagnosis ^1^	334	83		244	88		90	73	

Data are presented as median and 25th (P25) and 75th (P75) percentiles for continuous variables and as absolute and relative frequencies for categorical variables, respectively. ^1^ This variable combined the information obtained from all available follow-ups on the presence of selected diseases, such as cancer, osteoporosis, thyroid disease, diabetes, heart disease and chronic inflammatory bowel/liver/kidney diseases.

**Table 2:** Age-related changes in parameters of anthropometry and body composition before considering potential influencing factors (*n* = 401).

	Age		Age^2^	
	CE	(95% CI)	CE	(95% CI)
Body mass (kg)	−0.59	(−1.06, −0.13)	−1.64	(−2.09, −1.19)
Body mass index (kg/m^2^)	0.23	(0.06, 0.40)	−0.39	(−0.56, −0.23)
Upper arm circumference (cm)	−0.07	(−0.23, 0.08)	−0.09	(−0.24, 0.07)
Waist circumference (cm)	4.23	(3.75, 4.71)	−0.32	(−0.86, 0.21)
Fat mass (kg)	0.05	(−0.30, 0.39)	−0.92	(−1.28, −0.57)
Fat-free mass (kg)	−0.62	(−0.80, −0.44)	−0.64	(−0.82, −0.46)
Fat-free mass (%)	−0.40	(−0.65, −0.14)	0.42	(0.14, 0.70)

Abbreviations: CE, coefficient estimate; 95% CI, 95% confidence interval. Linear mixed-effects models with a linear and quadratic function of individual age (scaled to decades and centered around 7.2) as the only fixed effects. Data are presented as coefficient estimates and pointwise 95% confidence intervals.

**Table 3:** Predictors in linear mixed-effects models for anthropometric data (*n* = 401).

Predictor	Body Mass (kg)		Body Mass Index (kg/m^2^)		Upper Arm Circumference (cm)		Waist Circumference (cm)	
	CE	(95% CI)	CE	(95% CI)	CE	(95% CI)	CE	(95% CI)
Intercept	7.1 × 10^+1^ ***	(6.9 × 10^+1^, 7.3 × 10^+1^)	2.7 × 10^+1^ ***	(2.7 × 10^+1^, 2.8 × 10^+1^)	3.0 × 10^+1^ ***	(2.9 × 10^+1^, 3.0 × 10^+1^)	8.9 × 10^+1^ ***	(8.9 × 10^+1^, 9.0 × 10^+1^)
Age (years)	−4.8 × 10^−2^	(−1.3 × 10^−1^, 3.9 × 10^−2^)	3.2 × 10^−2^ *	(5.7 × 10^−4^, 6.4 × 10^−2^)	7.3 × 10^−3^	(−2.2 × 10^−2^, 3.7 × 10^−2^)	5.0 × 10^−1^ ***	(4.4 × 10^−1^, 5.6 × 10^−1^)
Age (years)^2^	−1.7 × 10^−1^ ***	(−2.5 × 10^−1^, −8.6 × 10^−2^)	−4.3 × 10^−2^ ***	(−7.3 × 10^−2^, −1.3 × 10^−2^)	−1.3 × 10^−2^	(−4.1 × 10^−2^, 1.4 × 10^−2^)	9.9 × 10^−2^ ***	(4.0 × 10^−2^, 1.6 × 10^−1^)
Male sex	9.9 × 10^+0^ ***	(6.2 × 10^+0^, 1.4 × 10^+1^)	−4.2 × 10^−1^	(−1.7 × 10^+0^, 8.9 × 10^−1^)	9.2 × 10^−2^	(−8.6 × 10^−1^, 1.0 × 10^+0^)	2.1 × 10^+0^ **	(3.6 × 10^−1^, 3.8 × 10^+0^)
PAI	−2.1 × 10^−1^	(−1.7 × 10^+0^, 1.3 × 10^+0^)	−1.0 × 10^−1^	(−6.5 × 10^−1^, 4.4 × 10^−1^)	7.7 × 10^−2^	(−6.7 × 10^−1^, 8.3 × 10^−1^)	−2.6 × 10^−1^	(−1.9 × 10^+0^, 1.4 × 10^+0^)
Protein intake (g/day)	−2.2 × 10^−3^	(−1.1 × 10^−2^, 7.1 × 10^−3^)	−1.3 × 10^−3^	(−4.7 × 10^−3^, 2.2 × 10^−3^)	1.9 × 10^−3^	(−2.9 × 10^−3^, 6.6 × 10^−3^)	−8.3 × 10^−3^	(−1.9 × 10^−2^, 2.0 × 10^−3^)
Year of entry (year)	8.5 × 10^−1^ **	(1.9 × 10^−1^, 1.5 × 10^+0^)	2.3 × 10^−1 ‡^	(−4.9 × 10^−3^, 4.7 × 10^−1^)	1.8 × 10^−1^ *	(1.2 × 10^−2^, 3.6 × 10^−1^)	1.3 × 10^−1^	(−1.7 × 10^−1^, 4.2 × 10^−1^)
Use of diuretics	−5.5 × 10^−2^	(−6.1 × 10^−1^, 5.0 × 10^−1^)	3.1 × 10^−2^	(−1.7 × 10^−1^, 2.4 × 10^−1^)	1.2 × 10^−1^	(−1.5 × 10^−1^, 3.9 × 10^−1^)	3.1 × 10^−1^	(−2.7 × 10^−1^, 9.0 × 10^−1^)
I (PAI:age)	−6.4 × 10^−2^	(−2.5 × 10^−1^, 1.2 × 10^−1^)	−3.2 × 10^−2^	(−1.0 × 10^−1^, 3.7 × 10^−2^)	−2.7 × 10^−2^	(−1.2 × 10^−1^, 6.4 × 10^−2^)	−1.2 × 10^−1^	(−3.1 × 10^−1^, 7.5 × 10^−2^)
I (protein intake:age)	−3.7 × 10^−4^	(−1.4 × 10^−3^, 6.8 × 10^−4^)	−1.4 × 10^−4^	(−5.3 × 10^−4^, 2.5 × 10^−4^)	2.2 × 10^−5^	(−4.9 × 10^−4^, 5.3 × 10^−4^)	−1.6 × 10^−4^	(−1.3 × 10^−3^, 9.5 × 10^−4^)
I (year of entry:age)	−1.9 × 10^−2^	(−4.7 × 10^−2^, 9.5 × 10^−3^)	−3.8 × 10^−3^	(−1.4 × 10^−2^, 6.4 × 10^−3^)	7.4 × 10^−3^	(−2.1 × 10^−3^, 1.7 × 10^−2^)	3.0 × 10^−2^ ***	(1.1 × 10^−2^, 5.0 × 10^−2^)
I (male sex:age)	−4.2 × 10^−3^	(−1.5 × 10^−1^, 1.4 × 10^−1^)	−1.6 × 10^−2^	(−7.0 × 10^−2^, 3.8 × 10^−2^)	−4.6 × 10^−2 ‡^	(−9.5 × 10^−2^, 2.7 × 10^−3^)	−9.8 × 10^−2^ *	(−2.0 × 10^−1^, −8.0 × 10^−4^)
I (male sex:age^2^)	−9.5 × 10^−3^	(−1.5 × 10^−1^, 1.3 × 10^−1^)	5.8 × 10^−3^	(−4.7 × 10^−2^, 5.9 × 10^−2^)	1.2 × 10^−2^	(−3.7 × 10^−2^, 6.0 × 10^−2^)	−9.5 × 10^−2^	(−2.0 × 10^−1^, 8.6 × 10^−3^)
I (male sex:PAI)	−8.4 × 10^−1^	(−3.7 × 10^+0^, 2.0 × 10^+0^)	−3.7 × 10^−1^	(−1.4 × 10^+0^, 6.7 × 10^−1^)	−5.4 × 10^−1^	(−2.0 × 10^+0^, 8.7 × 10^−1^)	−1.5 × 10^+0^	(−4.6 × 10^+0^, 1.5 × 10^+0^)
I (male sex:protein intake)	1.6 × 10^−3^	(−1.3 × 10^−2^, 1.6 × 10^−2^)	9.6 × 10^−4^	(−4.5 × 10^−3^, 6.5 × 10^−3^)	−1.8 × 10^−3^	(−9.3 × 10^−3^, 5.7 × 10^−3^)	2.8 × 10^−3^	(−1.4 × 10^−2^, 1.9 × 10^−2^)
Body mass (kg)							7.9 × 10^−1^ ***	(7.5 × 10^−1^, 8.4 × 10^−1^)

Abbreviations: CE, coefficient estimate; 95% CI, 95% confidence interval; PAI, physical activity index; I(a:b), interaction effect for a and b. Data are presented as coefficient estimates and 95% confidence intervals adjusted for simultaneous inference. ^‡^ *P* < 0.10 (indicating trend); * *P* < 0.05; ** *P* < 0.01; *** *P* < 0.001. Age^2^ denotes the quadratic function of individual age to allow curvilinear age-related changes in dependent variables.

**Table 4:** Predictors in linear mixed-effects models for body composition (*n* = 401).

Predictor	Fat Mass (kg)		Fat-Free Mass (kg)		Fat-Free Mass (%)	
	CE	(95% CI)	CE	(95% CI)	CE	(95% CI)
Intercept	3.1 × 10^+1^ ***	(2.9 × 10^+1^, 3.2 × 10^+1^)	4.0 × 10^+1^ ***	(3.9 × 10^+1^, 4.1 × 10^+1^)	5.7 × 10^+1^ ***	(5.6 × 10^+1^, 5.8 × 10^+1^)
Age (years)	7.9 × 10^−3^	(−5.7 × 10^−2^, 7.3 × 10^−2^)	−5.8 × 10^−2^ ***	(−9.2 × 10^−2^, −2.3 × 10^−2^)	−3.9 × 10^−2^	(−8.8 × 10^−2^, 9.5 × 10^−3^)
Age (years)^2^	−1.0 × 10^−1^ ***	(−1.6 × 10^−1^, −3.7 × 10^−2^)	−5.4 × 10^−2^ ***	(−8.6 × 10^−2^, −2.2 × 10^−2^)	4.1 × 10^−2^	(−8.6 × 10^−3^, 9.1 × 10^−2^)
Male sex	−5.7 × 10^+0^ ***	(−8.4 × 10^+0^, −3.1 × 10^+0^)	1.6 × 10^+1^ ***	(1.4 × 10^+1^, 1.7 × 10^+1^)	1.2 × 10^+1^ ***	(1.1 × 10^+1^, 1.4 × 10^+1^)
PAI	−3.1 × 10^−1^	(−1.6 × 10^+0^, 9.4 × 10^−1^)	4.9 × 10^−2^	(−7.3 × 10^−1^, 8.3 × 10^−1^)	2.0 × 10^−1^	(−9.1 × 10^−1^, 1.3 × 10^+0^)
Protein intake (g/day)	−3.0 × 10^−3^	(−1.1 × 10^−2^, 4.9 × 10^−3^)	1.6 × 10^−3^	(−3.4 × 10^−3^, 6.5 × 10^−3^)	2.0 × 10^−3^	(−5.0 × 10^−3^, 9.0 × 10^−3^)
Year of entry (year)	5.5 × 10^−1^ *	(7.7 × 10^−2^, 1.0 × 10^+0^)	2.5 × 10^−1^ *	(2.0 × 10^−2^, 4.9 × 10^−1^)	−2.3 × 10^−1^	(−5.3 × 10^−1^, 7.7 × 10^−2^)
Use of diuretics	2.0 × 10^−1^	(−2.7 × 10^−1^, 6.6 × 10^−1^)	−2.1 × 10^−1^	(−4.9 × 10^−1^, 7.9 × 10^−2^)	−3.8 × 10^−1 ‡^	(−7.9 × 10^−1^, 2.9 × 10^−2^)
I (PAI:age)	−1.9 × 10^−3^	(−1.6 × 10^−1^, 1.6 × 10^−1^)	−6.3 × 10^−2^	(−1.6 × 10^−1^, 3.3 × 10^−2^)	−5.1 × 10^−2^	(−1.9 × 10^−1^, 8.5 × 10^−2^)
I (protein intake:age)	−4.5 × 10^−4^	(−1.3 × 10^−3^, 4.4 × 10^−4^)	2.4 × 10^−5^	(−5.2 × 10^−4^, 5.7 × 10^−4^)	5.8 × 10^−4^	(−2.1 × 10^−4^, 1.4 × 10^−3^)
I (year of entry:age)	−4.5 × 10^−3^	(−2.6 × 10^−2^, 1.6 × 10^−2^)	−1.2 × 10^−2^ *	(−2.3 × 10^−2^, −1.0 × 10^−3^)	−5.5 × 10^−3^	(−2.1 × 10^−2^, 1.0 × 10^−2^)
I (male sex:age)	2.2 × 10^−3^	(−1.1 × 10^−1^, 1.1 × 10^−1^)	2.1 × 10^−3^	(−5.6 × 10^−2^, 6.0 × 10^−2^)	1.1 × 10^−2^	(−7.0 × 10^−2^, 9.3 × 10^−2^)
I (male sex:age^2^)	3.2 × 10^−2^	(−8.1 × 10^−2^, 1.4 × 10^−1^)	−4.5 × 10^−2^	(−1.0 × 10^−1^, 1.0 × 10^−2^)	−1.9 × 10^−2^	(−1.1 × 10^−1^, 6.8 × 10^−2^)
I (male sex:PAI)	−9.3 × 10^−1^	(−3.3 × 10^+0^, 1.5 × 10^+0^)	2.8 × 10^−1^	(−1.2 × 10^+0^, 1.8 × 10^+0^)	1.1 × 10^+0^	(−1.0 × 10^+0^, 3.2 × 10^+0^)
I (male sex:protein intake)	5.3 × 10^−4^	(−1.2 × 10^−2^, 1.3 × 10^−2^)	1.2 × 10^−3^	(−6.6 × 10^−3^, 9.0 × 10^−3^)	1.7 × 10^−3^	(−9.5 × 10^−3^, 1.3 × 10^−2^)

Abbreviations: CE, coefficient estimate; 95% CI, 95% confidence interval; PAI, physical activity index; I(a:b), interaction effect for a and b. Data are presented as coefficient estimates and 95% confidence intervals adjusted for simultaneous inference. ^‡^ *P* < 0.10 (indicating trend); * *P* < 0.05; *** *P* < 0.001. Age^2^ denotes the quadratic function of individual age to allow curvilinear age-related changes in dependent variables.

## Supplementary Materials

The following supplementary materials are available online at https://www.mdpi.com/2072-6643/12/12/3626/s1, Table S1: Predictors in linear mixed models for anthropometric data after exclusion of outlying observations; Table S2: Predictors in linear mixed models for body composition after exclusion of outlying observations; Table S3: Predictors in linear mixed models for anthropometric data after consideration of disease history (*n* = 401); Table S4: Predictors in linear mixed models for body composition after consideration of disease history (*n* = 401); Table S5: Predictors in linear mixed models for anthropometric data after consideration of energy intake instead of protein intake (*n* = 401); Table S6: Predictors in linear mixed models for body composition after consideration of energy intake instead of protein intake (*n* = 401); Table S7: Predictors in linear mixed models for anthropometric data in subjects with at least 7 follow-ups (*n* = 226); Table S8: Predictors in linear mixed models for body composition in subjects with at least 7 follow-ups (*n* = 226); Figure S1: Comparison of excluded with selected subjects based on MANOVA; Figure S2: Age-related changes in protein intake, energy intake and physical activity index separated by sex; Figure S3: Changes in absolute fat-free mass and waist circumference with increasing age in dependence of the year of entry (YoE).

## Abbreviations

95% CI	95% confidence interval
BIA	bioelectrical impedance analysis
BMI	body mass index
CE	coefficient estimate
FFM	fat-free mass
FM	fat mass
MJ	megajoule
GISELA	longitudinal study on nutrition and health status of senior citizens in Giessen
PAI	physical activity index
