# Supplementary material for: Trajectories of Body Composition during Advanced Aging in Consideration of Diet and Physical Activity: A 20-Year Longitudinal Study
Source: Nutrients. 2020 Nov 25;12(12):3626. doi: 10.3390/nu12123626 (PMC7761400; doi:10.3390/nu12123626)
Supplement: Supplementary file 1 [file nutrients-12-03626-s001.pdf]

Supplementary Table S1. Predictors in linear mixed models for anthropometric data after exclusion of outlying observations.

Predictor	Body mass (kg) (*n* = 399)		BMI (kg/m²) (*n* = 399)		UAC (cm) (*n* = 401)		WC (cm) (*n* = 401)	
	CE	*[95% CI]*	*CE*	*[95% CI]*	*CE*	*[95% CI]*	*CE*	*[95% CI]*
Intercept	7.1E+01 ***	[6.9E+01, 7.3E+01]	2.7E+01 ***	[2.7E+01, 2.8E+01]	3.0E+01 ***	[2.9E+01, 3.0E+01]	8.9E+01 ***	[8.9E+01, 9.0E+01]
Age (years)	-3.4E-02	[-1.2E-01, 5.4E-02]	3.7E-02 **	[6.2E-03, 6.9E-02]	8.2E-03	[-2.1E-02, 3.8E-02]	5.0E-01 ***	[4.4E-01, 5.6E-01]
Age (years)²	-1.6E-01 ***	[-2.3E-01, -8.1E-02]	-4.0E-02 ***	[-6.9E-02, -1.1E-02]	-1.2E-02	[-4.0E-02, 1.5E-02]	1.0E-01 ***	[4.1E-02, 1.6E-01]
Male sex	1.0E+01 ***	[6.4E+00, 1.4E+01]	-3.4E-01	[-1.7E+00, 9.7E-01]	9.4E-02	[-8.6E-01, 1.0E+00]	2.1E+00 **	[3.6E-01, 3.7E+00]
PAI	1.6E-01	[-1.2E+00, 1.5E+00]	-7.0E-03	[-5.2E-01, 5.1E-01]	8.1E-02	[-6.6E-01, 8.2E-01]	-4.7E-01	[-2.0E+00, 1.1E+00]
Protein intake (g/d)	2.0E-04	[-8.5E-03, 8.9E-03]	-1.8E-04	[-3.5E-03, 3.1E-03]	1.7E-03	[-2.9E-03, 6.4E-03]	-7.9E-03	[-1.8E-02, 2.0E-03]
Year of entry (year)	8.5E-01 **	[1.8E-01, 1.5E+00]	2.2E-01 ‡	[-1.9E-02, 4.6E-01]	1.8E-01 *	[7.9E-03, 3.5E-01]	1.3E-01	[-1.6E-01, 4.3E-01]
Use of diuretics	5.1E-02	[-4.7E-01, 5.7E-01]	4.9E-02	[-1.5E-01, 2.5E-01]	1.1E-01	[-1.6E-01, 3.8E-01]	2.8E-01	[-2.9E-01, 8.4E-01]
I (PAI:age)	-5.7E-02	[-2.3E-01, 1.2E-01]	-3.1E-02	[-9.7E-02, 3.5E-02]	-2.9E-02	[-1.2E-01, 6.1E-02]	-1.3E-01	[-3.1E-01, 6.3E-02]
I (protein intake:age)	-3.0E-04	[-1.3E-03, 6.9E-04]	-1.6E-04	[-5.3E-04, 2.2E-04]	4.9E-06	[-5.0E-04, 5.1E-04]	-2.4E-04	[-1.3E-03, 8.4E-04]
I (year of entry:age)	-1.9E-02	[-4.7E-02, 1.0E-02]	-4.1E-03	[-1.4E-02, 6.1E-03]	7.6E-03	[-1.9E-03, 1.7E-02]	3.3E-02 ***	[1.4E-02, 5.2E-02]
I (male sex:age)	-1.4E-02	[-1.7E-01, 1.4E-01]	-1.7E-02	[-7.0E-02, 3.7E-02]	-4.8E-02 ‡	[-9.7E-02, 9.6E-04]	-9.4E-02 ‡	[-1.9E-01, 1.8E-03]
I (male sex:age²)	-1.7E-02	[-1.5E-01, 1.2E-01]	6.2E-03	[-4.5E-02, 5.8E-02]	1.1E-02	[-3.8E-02, 5.9E-02]	-9.6E-02 ‡	[-2.0E-01, 8.1E-03]
I (male sex:PAI)	-1.5E+00	[-4.2E+00, 1.1E+00]	-5.3E-01	[-1.5E+00, 4.7E-01]	-5.8E-01	[-2.0E+00, 8.2E-01]	-1.3E+00	[-4.3E+00, 1.6E+00]
I (male sex:protein intake)	-1.1E-04	[-1.4E-02, 1.4E-02]	-6.1E-05	[-5.3E-03, 5.2E-03]	-1.3E-03	[-8.8E-03, 6.1E-03]	2.6E-03	[-1.3E-02, 1.8E-02]
Body mass (kg)							7.9E-01 ***	[7.5E-01, 8.4E-01]

Abbreviations: CE, coefficient estimate; 95% CI, 95% confidence interval; BMI, body mass index; UAC, upper arm circumference; WC, waist circumference; PAI, physical activity index; I(a:b), interaction effect for a and b. Data are presented as coefficient estimates and 95% confidence intervals adjusted for simultaneous inference. ‡ $P < 0.10$; * $P < 0.05$; ** $P < 0.01$; *** $P < 0.001$. Numbers are written in the scientific E notation.

Supplementary Table S2. Predictors in linear mixed models for body composition after exclusion of outlying observations.

Predictor	Fat mass (kg) (n = 400)		Fat-free mass (kg) (n = 401)		Fat-free mass (%) (n = 401)	
	CE	*[95% CI]*	*CE*	*[95% CI]*	*CE*	*[95% CI]*
Intercept	3.1E+01 ***	[2.9E+01, 3.2E+01]	4.0E+01 ***	[3.9E+01, 4.1E+01]	5.7E+01 ***	[5.6E+01, 5.8E+01]
Age (years)	1.9E-02	[-4.6E-02, 8.4E-02]	-6.0E-02 ***	[-9.4E-02, -2.7E-02]	-4.5E-02	[-9.1E-02, 1.8E-03]
Age (years)^2	-9.0E-02 ***	[-1.5E-01, -2.7E-02]	-5.7E-02 ***	[-8.9E-02, -2.6E-02]	3.7E-02 ‡	[-1.1E-02, 8.5E-02]
Male sex	-5.6E+00 ***	[-8.3E+00, -3.0E+00]	1.6E+01 ***	[1.4E+01, 1.7E+01]	1.2E+01 ***	[1.1E+01, 1.4E+01]
PAI	-2.3E-02	[-1.2E+00, 1.1E+00]	6.0E-02	[-6.6E-01, 7.8E-01]	2.3E-01	[-7.8E-01, 1.2E+00]
Protein intake (g/d)	-1.2E-03	[-8.6E-03, 6.2E-03]	2.0E-03	[-2.5E-03, 6.5E-03]	8.9E-04	[-5.6E-03, 7.3E-03]
Year of entry (year)	5.6E-01 **	[8.0E-02, 1.0E+00]	2.5E-01 *	[1.5E-02, 4.9E-01]	-2.3E-01	[-5.3E-01, 7.1E-02]
Use of diuretics	3.4E-01	[-1.0E-01, 7.8E-01]	-2.1E-01	[-4.8E-01, 5.0E-02]	-4.0E-01 *	[-7.7E-01, -2.3E-02]
I (PAI:age)	6.8E-03	[-1.4E-01, 1.5E-01]	-5.8E-02	[-1.5E-01, 3.0E-02]	-3.1E-02	[-1.6E-01, 9.5E-02]
I (protein intake:age)	-5.6E-04	[-1.4E-03, 2.8E-04]	6.4E-05	[-4.4E-04, 5.7E-04]	5.7E-04	[-1.5E-04, 1.3E-03]
I (year of entry:age)	-5.8E-03	[-2.7E-02, 1.5E-02]	-1.3E-02 **	[-2.3E-02, -1.9E-03]	-6.9E-03	[-2.2E-02, 8.1E-03]
I (male sex:age)	-4.2E-03	[-1.2E-01, 1.1E-01]	4.2E-03	[-5.1E-02, 6.0E-02]	1.3E-02	[-6.5E-02, 9.1E-02]
I (male sex:age^2)	2.8E-02	[-8.5E-02, 1.4E-01]	-4.4E-02	[-9.9E-02, 1.1E-02]	-2.5E-02	[-1.1E-01, 6.0E-02]
I (male sex:PAI)	-1.3E+00	[-3.6E+00, 9.0E-01]	2.2E-01	[-1.1E+00, 1.6E+00]	1.3E+00	[-6.8E-01, 3.2E+00]
I (male sex:protein intake)	-1.3E-03	[-1.3E-02, 1.0E-02]	7.5E-04	[-6.4E-03, 8.0E-03]	2.3E-03	[-8.0E-03, 1.3E-02]

Abbreviations: CE, coefficient estimate; 95% CI, 95% confidence interval; PAI, physical activity index; I(a:b), interaction effect for a and b. Data are presented as coefficient estimates and 95% confidence intervals adjusted for simultaneous inference. ‡ $P < 0.10$; * $P < 0.05$; ** $P < 0.01$; *** $P < 0.001$. Numbers are written in the scientific E notation.

Supplementary Table S3. Predictors in linear mixed models for anthropometric data after consideration of disease history (n = 401).

Predictor	Body mass (kg)		Body mass index (kg/m²)		Upper arm circumference (cm)		Waist circumference (cm)	
	CE	*[95% CI]*	*CE*	*[95% CI]*	*CE*	*[95% CI]*	*CE*	*[95% CI]*
Intercept	6.8E+01 ***	[6.4E+01, 7.3E+01]	2.7E+01 ***	[2.5E+01, 2.8E+01]	2.9E+01 ***	[2.8E+01, 3.1E+01]	8.9E+01 ***	[8.7E+01, 9.1E+01]
Age (years)	-3.3E-02	[-2.3E-01, 1.6E-01]	4.0E-02	[-3.2E-02, 1.1E-01]	1.3E-02	[-5.4E-02, 7.9E-02]	4.6E-01 ***	[3.3E-01, 5.9E-01]
Age (years)²	-1.7E-01 ***	[-2.5E-01, -8.5E-02]	-4.3E-02 ***	[-7.3E-02, -1.3E-02]	-1.3E-02	[-4.1E-02, 1.5E-02]	9.9E-02 ***	[3.9E-02, 1.6E-01]
Male sex	1.0E+01 ***	[6.5E+00, 1.4E+01]	-2.9E-01	[-1.6E+00, 1.0E+00]	1.1E-01	[-8.7E-01, 1.1E+00]	2.2E+00 **	[4.8E-01, 4.0E+00]
PAI	-2.1E-01	[-1.7E+00, 1.3E+00]	-1.1E-01	[-6.6E-01, 4.4E-01]	7.4E-02	[-6.8E-01, 8.3E-01]	-2.6E-01	[-1.9E+00, 1.4E+00]
Protein intake (g/d)	-2.2E-03	[-1.2E-02, 7.2E-03]	-1.3E-03	[-4.7E-03, 2.2E-03]	1.8E-03	[-2.9E-03, 6.6E-03]	-8.3E-03	[-1.9E-02, 2.0E-03]
Year of entry (year)	8.4E-01 **	[1.7E-01, 1.5E+00]	2.3E-01 ‡	[-1.2E-02, 4.6E-01]	1.8E-01 *	[8.9E-03, 3.6E-01]	1.2E-01	[-1.7E-01, 4.2E-01]
Use of diuretics	-5.8E-02	[-6.2E-01, 5.0E-01]	3.0E-02	[-1.8E-01, 2.4E-01]	1.2E-01	[-1.6E-01, 3.9E-01]	3.1E-01	[-2.8E-01, 9.0E-01]
I (PAI:age)	-6.4E-02	[-2.5E-01, 1.2E-01]	-3.2E-02	[-1.0E-01, 3.8E-02]	-2.7E-02	[-1.2E-01, 6.5E-02]	-1.2E-01	[-3.1E-01, 7.8E-02]
I (protein intake:age)	-3.7E-04	[-1.4E-03, 6.9E-04]	-1.4E-04	[-5.4E-04, 2.5E-04]	2.3E-05	[-4.9E-04, 5.4E-04]	-1.7E-04	[-1.3E-03, 9.6E-04]
I (year of entry:age)	-1.9E-02	[-4.7E-02, 9.9E-03]	-3.8E-03	[-1.4E-02, 6.5E-03]	7.4E-03	[-2.2E-03, 1.7E-02]	3.0E-02 ***	[1.1E-02, 5.0E-02]
I (male sex:age)	-6.0E-03	[-1.6E-01, 1.5E-01]	-1.7E-02	[-7.3E-02, 3.8E-02]	-4.7E-02 ‡	[-9.8E-02, 3.4E-03]	-9.0E-02	[-1.9E-01, 1.1E-02]
I (male sex:age²)	-9.9E-03	[-1.5E-01, 1.3E-01]	5.6E-03	[-4.8E-02, 5.9E-02]	1.2E-02	[-3.7E-02, 6.0E-02]	-9.6E-02	[-2.0E-01, 9.3E-03]
I (male sex:PAI)	-8.2E-01	[-3.7E+00, 2.0E+00]	-3.6E-01	[-1.4E+00, 6.9E-01]	-5.4E-01	[-2.0E+00, 8.9E-01]	-1.5E+00	[-4.6E+00, 1.6E+00]
I (male sex:protein intake)	1.6E-03	[-1.3E-02, 1.7E-02]	9.7E-04	[-4.6E-03, 6.5E-03]	-1.8E-03	[-9.4E-03, 5.7E-03]	2.9E-03	[-1.4E-02, 1.9E-02]
Body mass (kg)							7.9E-01 ***	[7.5E-01, 8.4E-01]
I (disease diagnosis:age)	-1.7E-02	[-2.1E-01, 1.8E-01]	-9.4E-03	[-8.1E-02, 6.2E-02]	-6.1E-03	[-7.2E-02, 6.0E-02]	4.6E-02	[-8.4E-02, 1.8E-01]
Disease diagnosis	2.7E+00	[-1.8E+00, 7.3E+00]	9.0E-01	[-7.1E-01, 2.5E+00]	1.3E-01	[-1.0E+00, 1.3E+00]	1.0E+00	[-9.8E-01, 3.1E+00]

Abbreviations: CE, coefficient estimate; 95% CI, 95% confidence interval; PAI, physical activity index; I(a:b), interaction effect for a and b. Data are presented as coefficient estimates and 95% confidence intervals adjusted for simultaneous inference. ‡ $P < 0.10$; * $P < 0.05$; ** $P < 0.01$; *** $P < 0.001$. Numbers are written in the scientific E notation.

Supplementary Table S4. Predictors in linear mixed models for body composition after consideration of disease history ($n = 401$).

Predictor	Fat mass (kg)		Fat-free mass (kg)		Fat-free mass (%)	
	CE	*[95% CI]*	*CE*	*[95% CI]*	*CE*	*[95% CI]*
Intercept	2.9E+01 ***	[2.6E+01, 3.2E+01]	3.9E+01 ***	[3.8E+01, 4.1E+01]	5.8E+01 ***	[5.6E+01, 6.0E+01]
Age (years)	4.0E-02	[-1.1E-01, 1.9E-01]	-8.0E-02 *	[-1.6E-01, -2.2E-03]	-6.2E-02	[-1.7E-01, 4.7E-02]
Age (years)²	-1.0E-01 ***	[-1.6E-01, -3.6E-02]	-5.4E-02 ***	[-8.6E-02, -2.2E-02]	4.1E-02	[-9.4E-03, 9.1E-02]
Male sex	-5.4E+00 ***	[-8.2E+00, -2.7E+00]	1.6E+01 ***	[1.4E+01, 1.7E+01]	1.2E+01 ***	[1.0E+01, 1.4E+01]
PAI	-3.2E-01	[-1.6E+00, 9.4E-01]	5.0E-02	[-7.4E-01, 8.4E-01]	2.2E-01	[-9.1E-01, 1.3E+00]
Protein intake (g/d)	-3.0E-03	[-1.1E-02, 4.9E-03]	1.5E-03	[-3.4E-03, 6.5E-03]	2.0E-03	[-5.1E-03, 9.1E-03]
Year of entry (year)	5.4E-01 *	[6.2E-02, 1.0E+00]	2.5E-01 *	[1.3E-02, 4.9E-01]	-2.2E-01	[-5.2E-01, 8.7E-02]
Use of diuretics	1.9E-01	[-2.8E-01, 6.6E-01]	-2.1E-01	[-5.0E-01, 7.9E-02]	-3.8E-01	[-7.9E-01, 3.7E-02]
I (PAI:age)	-1.8E-03	[-1.6E-01, 1.6E-01]	-6.2E-02	[-1.6E-01, 3.5E-02]	-5.1E-02	[-1.9E-01, 8.7E-02]
I (protein intake:age)	-4.5E-04	[-1.4E-03, 4.5E-04]	2.3E-05	[-5.3E-04, 5.7E-04]	5.8E-04	[-2.1E-04, 1.4E-03]
I (year of entry:age)	-4.5E-03	[-2.6E-02, 1.7E-02]	-1.2E-02 *	[-2.3E-02, -9.4E-04]	-5.4E-03	[-2.1E-02, 1.0E-02]
I (male sex:age)	-2.5E-03	[-1.2E-01, 1.1E-01]	6.3E-03	[-5.3E-02, 6.6E-02]	1.5E-02	[-6.9E-02, 9.9E-02]
I (male sex:age²)	3.1E-02	[-8.2E-02, 1.4E-01]	-4.5E-02	[-1.0E-01, 1.1E-02]	-1.9E-02	[-1.1E-01, 7.0E-02]
I (male sex:PAI)	-9.0E-01	[-3.3E+00, 1.5E+00]	2.9E-01	[-1.2E+00, 1.8E+00]	1.1E+00	[-1.1E+00, 3.2E+00]
I (male sex:protein intake)	5.1E-04	[-1.2E-02, 1.3E-02]	1.2E-03	[-6.7E-03, 9.1E-03]	1.7E-03	[-9.6E-03, 1.3E-02]
I (disease diagnosis:age)	-3.7E-02	[-1.8E-01, 1.1E-01]	2.5E-02	[-5.2E-02, 1.0E-01]	2.6E-02	[-8.1E-02, 1.3E-01]
Disease diagnosis	2.0E+00	[-1.2E+00, 5.3E+00]	8.7E-01	[-7.3E-01, 2.5E+00]	-1.2E+00	[-3.3E+00, 8.8E-01]

Abbreviations: CE, coefficient estimate; 95% CI, 95% confidence interval; PAI, physical activity index; I(a:b), interaction effect for a and b. Data are presented as coefficient estimates and 95% confidence intervals adjusted for simultaneous inference. ‡ $P < 0.10$; * $P < 0.05$; ** $P < 0.01$; *** $P < 0.001$. Numbers are written in the scientific E notation.

Supplementary Table S5. Predictors in linear mixed models for anthropometric data after consideration of energy intake instead of protein intake ($n = 401$).

Predictor	Body mass (kg)		Body mass index (kg/m²)		Upper arm circumference (cm)		Waist circumference (cm)	
	CE	*[95% CI]*	*CE*	*[95% CI]*	*CE*	*[95% CI]*	*CE*	*[95% CI]*
Intercept	7.1E+01 ***	[6.9E+01, 7.3E+01]	2.7E+01 ***	[2.7E+01, 2.8E+01]	3.0E+01 ***	[2.9E+01, 3.0E+01]	8.9E+01 ***	[8.8E+01, 9.0E+01]
Age (years)	-5.5E-02	[-1.4E-01, 3.1E-02]	3.0E-02 ‡	[-1.7E-03, 6.1E-02]	9.4E-03	[-2.0E-02, 3.8E-02]	5.0E-01 ***	[4.4E-01, 5.5E-01]
Age (years)²	-1.7E-01 ***	[-2.5E-01, -8.8E-02]	-4.4E-02 ***	[-7.4E-02, -1.4E-02]	-1.3E-02	[-4.1E-02, 1.4E-02]	9.6E-02 ***	[3.7E-02, 1.6E-01]
Male sex	9.9E+00 ***	[6.2E+00, 1.4E+01]	-4.0E-01	[-1.7E+00, 9.0E-01]	4.8E-02	[-8.9E-01, 9.9E-01]	2.1E+00 **	[4.5E-01, 3.8E+00]
PAI	-2.2E-01	[-1.7E+00, 1.2E+00]	-1.1E-01	[-6.5E-01, 4.3E-01]	8.5E-02	[-6.6E-01, 8.3E-01]	-3.0E-01	[-1.9E+00, 1.3E+00]
Energy intake (MJ/d)	-2.6E-02	[-1.2E-01, 7.2E-02]	-1.4E-02	[-5.1E-02, 2.3E-02]	1.5E-02	[-3.5E-02, 6.5E-02]	-3.8E-02	[-1.5E-01, 7.1E-02]
Year of entry (year)	8.5E-01 **	[1.9E-01, 1.5E+00]	2.3E-01 ‡	[-5.1E-03, 4.7E-01]	1.8E-01 *	[1.1E-02, 3.6E-01]	1.3E-01	[-1.7E-01, 4.3E-01]
Use of diuretics	-5.9E-02	[-6.1E-01, 5.0E-01]	2.8E-02	[-1.8E-01, 2.3E-01]	1.2E-01	[-1.5E-01, 3.9E-01]	3.1E-01	[-2.8E-01, 8.9E-01]
I (PAI:age)	-7.0E-02	[-2.6E-01, 1.2E-01]	-3.5E-02	[-1.0E-01, 3.4E-02]	-2.8E-02	[-1.2E-01, 6.2E-02]	-1.2E-01	[-3.2E-01, 7.2E-02]
I (energy intake:age)	-1.6E-03	[-1.3E-02, 9.7E-03]	-2.2E-04	[-4.4E-03, 4.0E-03]	1.5E-03	[-3.9E-03, 7.0E-03]	-1.0E-03	[-1.3E-02, 1.1E-02]
I (year of entry:age)	-1.8E-02	[-4.7E-02, 9.8E-03]	-3.7E-03	[-1.4E-02, 6.6E-03]	7.6E-03	[-1.9E-03, 1.7E-02]	3.0E-02 ***	[1.1E-02, 4.9E-02]
I (male sex:age)	-5.1E-03	[-1.5E-01, 1.4E-01]	-1.7E-02	[-7.1E-02, 3.7E-02]	-4.8E-02 ‡	[-9.7E-02, 1.1E-03]	-9.9E-02 *	[-2.0E-01, -1.2E-03]
I (male sex:age²)	-8.6E-03	[-1.5E-01, 1.3E-01]	6.4E-03	[-4.6E-02, 5.9E-02]	1.2E-02	[-3.7E-02, 6.0E-02]	-9.4E-02	[-2.0E-01, 1.0E-02]
I (male sex:PAI)	-8.4E-01	[-3.7E+00, 2.0E+00]	-3.7E-01	[-1.4E+00, 6.7E-01]	-5.7E-01	[-2.0E+00, 8.5E-01]	-1.5E+00	[-4.5E+00, 1.6E+00]
I (male sex:energy intake)	3.8E-02	[-1.2E-01, 2.0E-01]	1.6E-02	[-4.4E-02, 7.6E-02]	-9.1E-03	[-9.1E-02, 7.3E-02]	-2.9E-02	[-2.1E-01, 1.5E-01]
Body mass (kg)							7.9E-01 ***	[7.5E-01, 8.4E-01]

Abbreviations: CE, coefficient estimate; 95% CI, 95% confidence interval; PAI, physical activity index; I(a:b), interaction effect for a and b. Data are presented as coefficient estimates and 95% confidence intervals adjusted for simultaneous inference. ‡ $P < 0.10$; * $P < 0.05$; ** $P < 0.01$; *** $P < 0.001$. Numbers are written in the scientific E notation.

Supplementary Table S6. Predictors in linear mixed models for body composition after consideration of energy intake instead of protein intake (n = 401).

Predictor	Fat mass (kg)		Fat-free mass (kg)		Fat-free mass (%)	
	CE	*[95% CI]*	*CE*	*[95% CI]*	*CE*	*[95% CI]*
Intercept	3.1E+01 ***	[2.9E+01, 3.2E+01]	4.0E+01 ***	[3.9E+01, 4.1E+01]	5.7E+01 ***	[5.6E+01, 5.8E+01]
Age (years)	-3.7E-03	[-6.8E-02, 6.1E-02]	-5.5E-02 ***	[-8.9E-02, -2.1E-02]	-2.5E-02	[-7.2E-02, 2.3E-02]
Age (years)^2	-1.0E-01 ***	[-1.7E-01, -3.9E-02]	-5.4E-02 ***	[-8.6E-02, -2.3E-02]	4.3E-02	[-6.8E-03, 9.2E-02]
Male sex	-5.7E+00 ***	[-8.4E+00, -3.1E+00]	1.6E+01 ***	[1.4E+01, 1.7E+01]	1.2E+01 ***	[1.1E+01, 1.4E+01]
PAI	-3.3E-01	[-1.6E+00, 9.2E-01]	5.5E-02	[-7.2E-01, 8.3E-01]	2.2E-01	[-9.0E-01, 1.3E+00]
Energy intake (MJ/d)	-3.3E-02	[-1.2E-01, 5.1E-02]	1.3E-02	[-3.9E-02, 6.5E-02]	3.2E-02	[-4.3E-02, 1.1E-01]
Year of entry (year)	5.5E-01 *	[7.7E-02, 1.0E+00]	2.5E-01 *	[2.0E-02, 4.9E-01]	-2.3E-01	[-5.3E-01, 7.7E-02]
Use of diuretics	2.0E-01	[-2.7E-01, 6.6E-01]	-2.1E-01	[-4.9E-01, 7.8E-02]	-3.8E-01 ‡	[-7.9E-01, 3.2E-02]
I (PAI:age)	-4.6E-03	[-1.6E-01, 1.5E-01]	-6.6E-02	[-1.6E-01, 3.0E-02]	-5.0E-02	[-1.9E-01, 8.7E-02]
I (energy intake:age)	-4.3E-03	[-1.4E-02, 5.2E-03]	1.9E-03	[-3.9E-03, 7.6E-03]	5.6E-03	[-2.7E-03, 1.4E-02]
I (year of entry:age)	-4.6E-03	[-2.6E-02, 1.6E-02]	-1.2E-02 *	[-2.3E-02, -7.8E-04]	-5.4E-03	[-2.1E-02, 1.0E-02]
I (male sex:age)	2.9E-03	[-1.1E-01, 1.1E-01]	1.3E-03	[-5.7E-02, 5.9E-02]	1.1E-02	[-7.0E-02, 9.3E-02]
I (male sex:age^2)	3.2E-02	[-8.1E-02, 1.4E-01]	-4.5E-02	[-1.0E-01, 1.1E-02]	-1.9E-02	[-1.1E-01, 6.9E-02]
I (male sex:PAI)	-9.0E-01	[-3.3E+00, 1.5E+00]	2.5E-01	[-1.2E+00, 1.7E+00]	1.0E+00	[-1.1E+00, 3.2E+00]
I (male sex:energy intake)	2.2E-02	[-1.2E-01, 1.6E-01]	1.9E-02	[-6.7E-02, 1.0E-01]	3.5E-04	[-1.2E-01, 1.2E-01]

Abbreviations: CE, coefficient estimate; 95% CI, 95% confidence interval; PAI, physical activity index; I(a:b), interaction effect for a and b. Data are presented as coefficient estimates and 95% confidence intervals adjusted for simultaneous inference. ‡ $P < 0.10$; * $P < 0.05$; ** $P < 0.01$; *** $P < 0.001$. Numbers are written in the scientific E notation.

Supplementary Table S7. Predictors in linear mixed models for anthropometric data in subjects with at least 7 follow-ups (n = 226).

Predictor	Body mass (kg)		Body mass index (kg/m²)		Upper arm circumference (cm)		Waist circumference (cm)	
	CE	*[95% CI]*	*CE*	*[95% CI]*	*CE*	*[95% CI]*	*CE*	*[95% CI]*
Intercept	7.1E+01 ***	[6.8E+01, 7.4E+01]	2.7E+01 ***	[2.6E+01, 2.8E+01]	3.0E+01 ***	[2.9E+01, 3.0E+01]	9.0E+01 ***	[8.9E+01, 9.1E+01]
Age (years)	-2.0E-02	[-1.2E-01, 7.7E-02]	4.2E-02 **	[6.1E-03, 7.7E-02]	2.3E-02	[-9.4E-03, 5.6E-02]	5.3E-01 ***	[4.6E-01, 6.0E-01]
Age (years)²	-2.1E-01 ***	[-3.0E-01, -1.2E-01]	-5.5E-02 ***	[-8.9E-02, -2.1E-02]	-1.7E-02	[-4.6E-02, 1.2E-02]	1.1E-01 ***	[4.5E-02, 1.8E-01]
Male sex	1.0E+01 ***	[5.6E+00, 1.5E+01]	-2.8E-01	[-1.9E+00, 1.3E+00]	1.1E-01	[-1.0E+00, 1.3E+00]	1.7E+00	[-4.7E-01, 3.9E+00]
PAI	1.1E-01	[-1.6E+00, 1.8E+00]	6.3E-02	[-5.7E-01, 7.0E-01]	5.2E-02	[-8.4E-01, 9.4E-01]	-1.2E-01	[-2.1E+00, 1.9E+00]
Protein intake (g/d)	-2.2E-03	[-1.4E-02, 9.2E-03]	-1.4E-03	[-5.6E-03, 2.9E-03]	8.4E-04	[-5.1E-03, 6.7E-03]	-1.0E-02	[-2.4E-02, 3.2E-03]
Year of entry (year)	1.1E+00 **	[2.4E-01, 2.0E+00]	2.7E-01	[-3.0E-02, 5.8E-01]	2.5E-01 *	[2.7E-02, 4.7E-01]	1.1E-01	[-3.0E-01, 5.1E-01]
Use of diuretics	-5.7E-02	[-6.9E-01, 5.7E-01]	2.6E-02	[-2.1E-01, 2.6E-01]	9.2E-02	[-2.2E-01, 4.0E-01]	2.8E-01	[-4.2E-01, 9.7E-01]
I (PAI:age)	-6.6E-02	[-2.7E-01, 1.4E-01]	-2.9E-02	[-1.1E-01, 4.8E-02]	-3.1E-02	[-1.3E-01, 7.0E-02]	-9.1E-02	[-3.2E-01, 1.3E-01]
I (protein intake:age)	-3.5E-04	[-1.6E-03, 8.6E-04]	-1.7E-04	[-6.2E-04, 2.7E-04]	-8.9E-05	[-6.7E-04, 4.9E-04]	-2.9E-04	[-1.6E-03, 1.0E-03]
I (year of entry:age)	-1.8E-02	[-5.2E-02, 1.5E-02]	-3.5E-03	[-1.6E-02, 8.6E-03]	1.1E-02 *	[3.1E-04, 2.3E-02]	2.9E-02 **	[5.4E-03, 5.2E-02]
I (male sex:age)	2.1E-02	[-1.4E-01, 1.8E-01]	-4.5E-03	[-6.4E-02, 5.5E-02]	-3.8E-02	[-9.0E-02, 1.5E-02]	-1.1E-01 ‡	[-2.2E-01, 4.1E-04]
I (male sex:age²)	2.0E-02	[-1.4E-01, 1.8E-01]	1.6E-02	[-4.3E-02, 7.5E-02]	2.0E-02	[-2.9E-02, 6.9E-02]	-1.1E-01	[-2.2E-01, 9.1E-03]
I (male sex:PAI)	-1.0E+00	[-4.2E+00, 2.2E+00]	-4.6E-01	[-1.6E+00, 7.1E-01]	-5.6E-01	[-2.2E+00, 1.0E+00]	-1.4E+00	[-5.0E+00, 2.2E+00]
I (male sex:protein intake)	1.0E-03	[-1.7E-02, 1.9E-02]	1.0E-03	[-5.5E-03, 7.5E-03]	-1.5E-03	[-1.0E-02, 7.6E-03]	8.2E-03	[-1.2E-02, 2.9E-02]
Body mass (kg)							7.9E-01 ***	[7.4E-01, 8.5E-01]

Abbreviations: CE, coefficient estimate; 95% CI, 95% confidence interval; PAI, physical activity index; I(a:b), interaction effect for a and b. Data are presented as coefficient estimates and 95% confidence intervals adjusted for simultaneous inference. ‡ $P < 0.10$; * $P < 0.05$; ** $P < 0.01$; *** $P < 0.001$. Numbers are written in the scientific E notation.

Supplementary Table S8. Predictors in linear mixed models for body composition in subjects with at least 7 follow-ups (*n* = 226).

Predictor	Fat mass (kg)		Fat-free mass (kg)		Fat-free mass (%)	
	CE	*[95% CI]*	*CE*	*[95% CI]*	*CE*	*[95% CI]*
Intercept	3.1E+01 ***	[2.9E+01, 3.3E+01]	4.0E+01 ***	[3.9E+01, 4.1E+01]	5.7E+01 ***	[5.6E+01, 5.8E+01]
Age (years)	3.1E-02	[-4.2E-02, 1.0E-01]	-5.0E-02 **	[-9.1E-02, -8.7E-03]	-5.1E-02 ‡	[-1.1E-01, 4.2E-03]
Age (years)^2	-1.3E-01 ***	[-2.0E-01, -5.3E-02]	-6.8E-02 ***	[-1.0E-01, -3.1E-02]	5.0E-02	[-4.6E-03, 1.0E-01]
Male sex	-5.7E+00 ***	[-9.0E+00, -2.3E+00]	1.6E+01 ***	[1.4E+01, 1.8E+01]	1.2E+01 ***	[1.0E+01, 1.5E+01]
PAI	-5.8E-02	[-1.5E+00, 1.4E+00]	1.5E-01	[-8.1E-01, 1.1E+00]	6.5E-02	[-1.3E+00, 1.4E+00]
Protein intake (g/d)	-3.5E-03	[-1.3E-02, 6.4E-03]	1.8E-03	[-4.6E-03, 8.2E-03]	2.9E-03	[-6.1E-03, 1.2E-02]
Year of entry (year)	7.1E-01 *	[9.0E-02, 1.3E+00]	3.1E-01 ‡	[-1.6E-02, 6.4E-01]	-3.1E-01	[-7.2E-01, 9.8E-02]
Use of diuretics	2.0E-01	[-3.4E-01, 7.4E-01]	-2.0E-01	[-5.4E-01, 1.4E-01]	-3.6E-01	[-8.4E-01, 1.2E-01]
I (PAI:age)	1.1E-02	[-1.7E-01, 1.9E-01]	-7.3E-02	[-1.9E-01, 3.9E-02]	-7.6E-02	[-2.3E-01, 8.0E-02]
I (protein intake:age)	-5.3E-04	[-1.6E-03, 5.0E-04]	1.1E-04	[-5.4E-04, 7.6E-04]	6.7E-04	[-2.5E-04, 1.6E-03]
I (year of entry:age)	-3.1E-03	[-2.8E-02, 2.2E-02]	-1.2E-02	[-2.6E-02, 2.3E-03]	-7.1E-03	[-2.6E-02, 1.2E-02]
I (male sex:age)	5.0E-03	[-1.2E-01, 1.3E-01]	1.5E-02	[-5.3E-02, 8.3E-02]	1.2E-02	[-7.8E-02, 1.0E-01]
I (male sex:age^2)	5.2E-02	[-7.3E-02, 1.8E-01]	-4.1E-02	[-1.0E-01, 2.1E-02]	-3.5E-02	[-1.3E-01, 5.9E-02]
I (male sex:PAI)	-1.2E+00	[-3.9E+00, 1.5E+00]	4.2E-01	[-1.3E+00, 2.2E+00]	1.2E+00	[-1.3E+00, 3.6E+00]
I (male sex:protein intake)	1.5E-03	[-1.4E-02, 1.7E-02]	2.0E-04	[-9.6E-03, 1.0E-02]	-5.6E-04	[-1.4E-02, 1.3E-02]

Abbreviations: CE, coefficient estimate; 95% CI, 95% confidence interval; PAI, physical activity index; I(a:b), interaction effect for a and b. Data are presented as coefficient estimates and 95% confidence intervals adjusted for simultaneous inference. ‡ $P < 0.10$; * $P < 0.05$; ** $P < 0.01$; *** $P < 0.001$. Numbers are written in the scientific E notation.

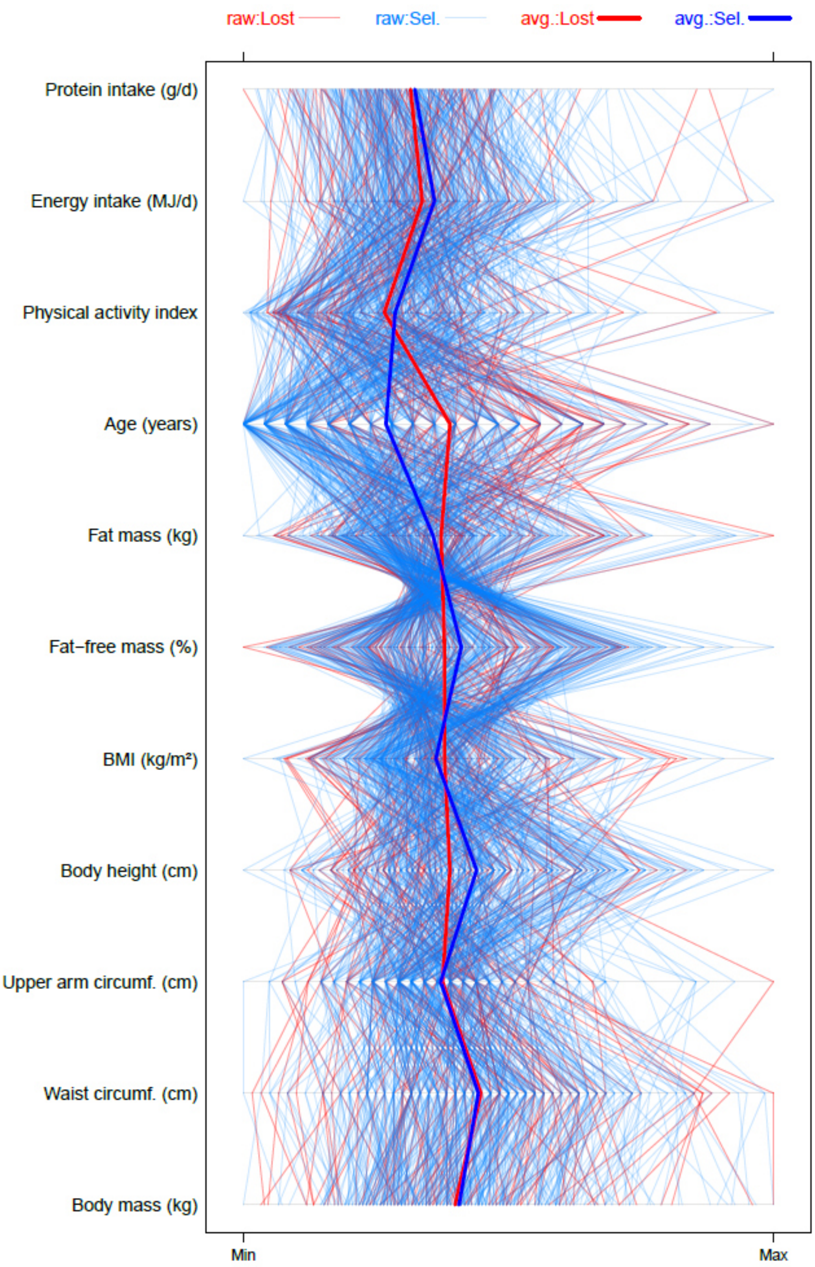

Supplementary Figure S1. Comparison of excluded with selected subjects based on MANOVA. This figure shows superposed parallel plots of raw data for body mass, waist circumference, upper arm circumference, body height, body mass index (BMI), relative fat-free mass, absolute fat mass, age, physical activity index, energy intake and protein intake, which were analyzed by MANOVA to compare excluded subjects ($n = 97$, colored in red) with the selected sample ($n = 401$, colored in blue) at baseline. The parallel plots are univariate scatterplots of all variables stacked parallel to each other, with values which belong to the same individual being linked by line segments forming a polyline. The average values of the variables within each group (i.e., across individuals of the pertaining group) are presented as respectively color-coded, thicker polylines. Before plotting, each variable was scaled individually, so the common scale was denoted with minimum and maximum of the variable rather than concrete ranges.

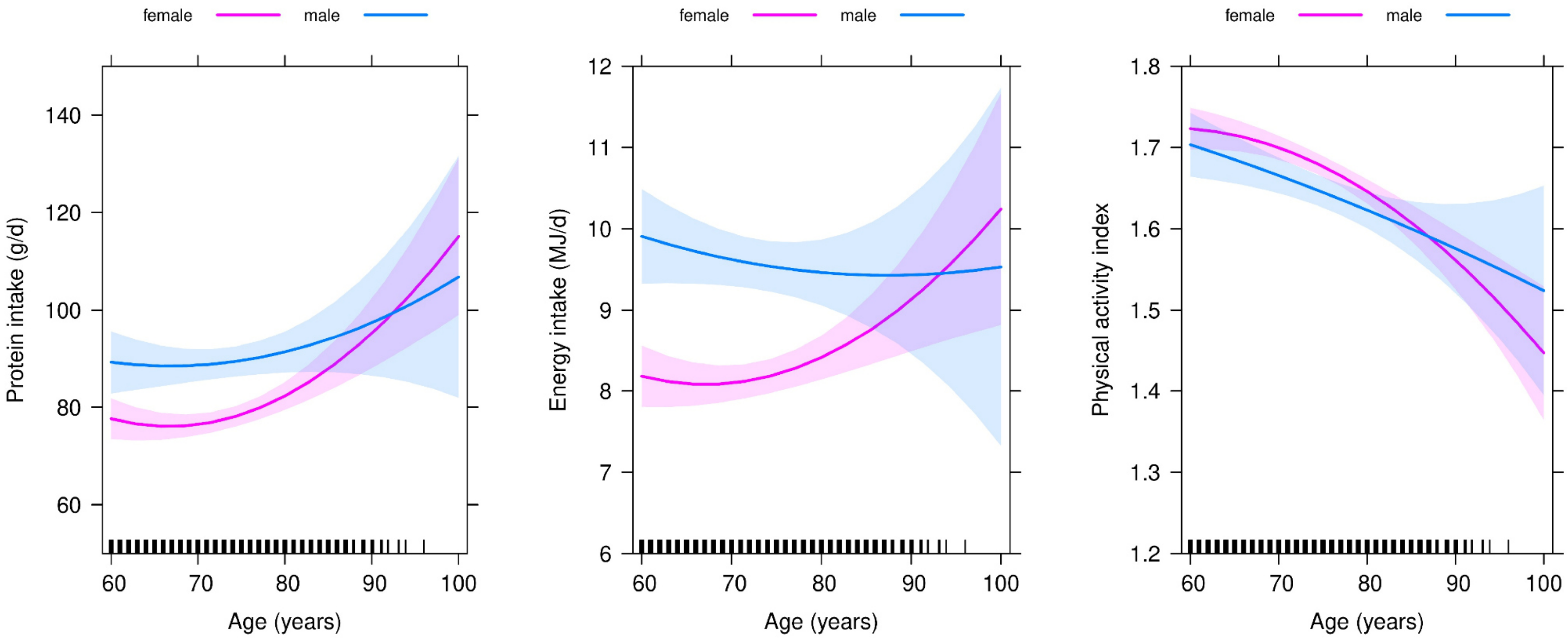

Supplementary Figure S2. Age-related changes in protein intake, energy intake and physical activity index separated by sex. This figure illustrates the effects of age and sex on protein intake, energy intake and physical activity index (n = 401). The thick lines represent the estimated means and the respective colored areas reflect the 95% confidence intervals. The associations are illustrated in magenta and blue color for female (n = 278) and male (n = 123) subjects, respectively. The small black pillars on the x-axis reflect the numbers of records at the respective age of the subjects.

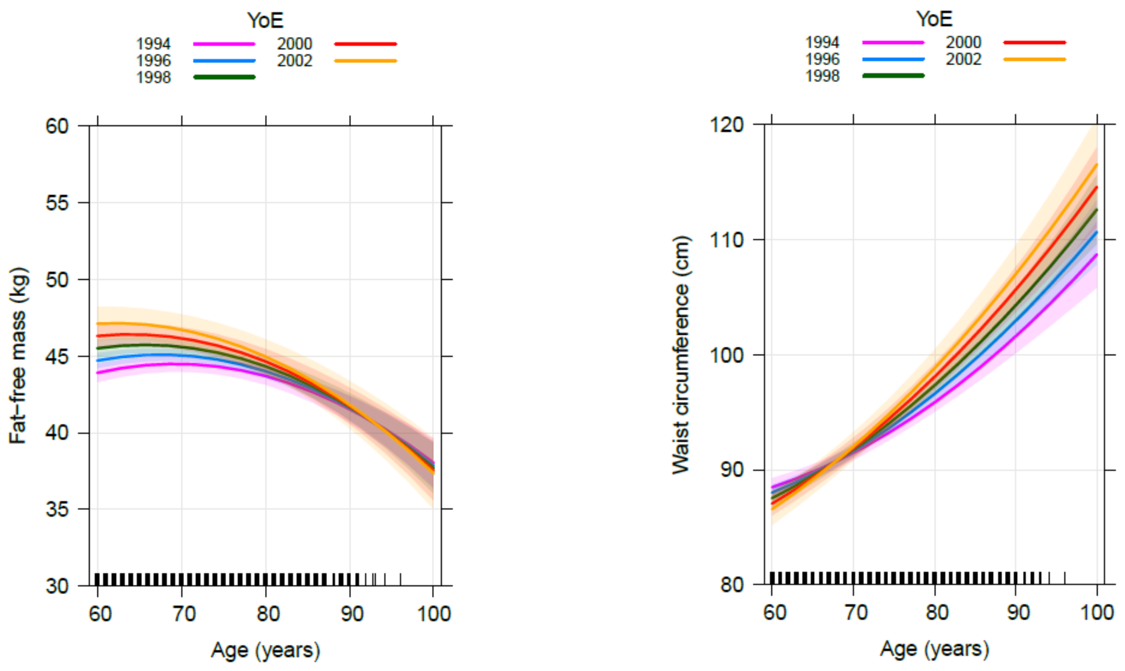

Supplementary Figure S3. Changes in absolute fat-free mass and waist circumference with increasing age in dependence of the year of entry (YoE). This figure displays the effect of age on absolute fat-free mass and waist circumference after controlling for other variables in the linear mixed-effects models separated by the first year of investigation (*n* = 401). The thick lines represent the estimated means and the respective colored areas reflect the 95% confidence intervals. The small black pillars on the x-axis reflect the numbers of records at the respective age of the subjects.
